# Stress Due to Inflation and Its Association with Anxiety and Depression Among Working-Age Adults in the United States

**DOI:** 10.3390/ijerph22010026

**Published:** 2024-12-29

**Authors:** Mona Pathak, Sophie Mitra, Jahnavi Pinnamraju, Patricia A. Findley, R. Constance Wiener, Hao Wang, Bo Zhou, Chan Shen, Usha Sambamoorthi

**Affiliations:** 1 Pharmacotherapy Department, College of Pharmacy, University of North Texas Health Sciences Center, Fort Worth, TX 76107, USAbo.zhou@unthsc.edu (B.Z.); usha.sambamoorthi@unthsc.edu (U.S.); 2 Department of Economics, Fordham University, 441 East Fordham Road, Bronx NY 10458, USA; 3 School of Social Work, Loyola University Chicago, 820 N. Michigan Ave., Chicago, IL 60611, USA; 4 Department of Dental Practice and Rural Health, School of Dentistry, 104A HSC Addition, West Virginia University, P.O. Box 9448, Morgantown, WV 26506, USA; 5 Department of Emergency Medicine, Integrative Emergency Services, JPS Health Network, Fort Worth, TX 76104, USA; 6 Department of Surgery, Penn State College of Medicine, Pennsylvania State University, Hershey, PA 17033, USA

**Keywords:** stress due to inflation, depression, anxiety, Household Pulse Survey, working-age adults

## Abstract

Inflation generates stress, which may lead to high rates of anxiety and depression. Given the recent surge and subsequent decline in the inflation rate in the United States, the prevalence of stress due to inflation may vary, as well as its relationship with anxiety and depression. Therefore, we investigated the prevalence of stress due to inflation and its association with anxiety and depression over time among working-age adults in the United States. We conducted a repeated cross-sectional analysis using Household Pulse Survey (HPS) data for the following weeks: Week 50 (5–17 October 2022) and Week 57 (26 April–8 May 2023). The HPS includes questions about individuals’ stress levels due to price increases in the past two months. We used logistic regressions to examine the association of stress (moderate or high stress versus little or no stress) due to inflation with depression and anxiety among working-age adults controlling for several factors, including demographic factors and social determinants of health. From October 2022 to April–May 2023, the prevalence of stress due to inflation affected more than three quarters of the population (77.7% and 78.7%, respectively). In logistic regressions, we found a significant positive association of stress due to inflation with depression (adjusted odds ratio (AOR) [95% CI] = 2.22 [1.92, 2.57]) and anxiety (AOR [95% CI] = 2.50 [2.18, 2.86]). Despite a decline in the prevalence of both depression and anxiety by three percentage points over the study period, the associations between stress, due to inflation on the one hand, and anxiety and depression, on the other, persisted over time. Stress due to inflation affects more than three-quarters of Americans, and is significantly associated with depression and anxiety. Stress due to inflation is a significant and persistent public health issue.

## 1. Introduction

Prior to the COVID-19 pandemic, inflation, a key economic indicator to measure purchasing power, had remained low (under 5%) in the United States (US) for nearly 40 years, except for a brief period around 1990 [1]. However, during the pandemic, it steadily increased, with a substantial negative impact on the economy, individuals, and families. It is well established that, as inflation increases, the costs of essential expenditures tend to surpass income growth, leading to higher financial demands and diminished purchasing power [2]. For example, what could be purchased with $100 in 2019 required approximately $115 by 2022 [3]. Such a loss in purchasing power can exacerbate financial hardships, as well as stress, for many households already affected by COVID-19 [4]. Indeed, a high prevalence of stress due to inflation has been reported in newspaper reports and in recent articles using data from the Census Household Pulse Survey (HPS) [5,6,7,8].

Furthermore, prolonged stress can lead to poor mental health [9]. Researchers have investigated the impact of stress, specifically financial stress, on mental health, albeit under different circumstances. For example, financial worries have been found to be associated with higher levels of psychological distress [10]. A systematic review reported that financial stress was associated with an increased risk of depressive symptoms among adults [11]. While the impact of economic instability on mental health has been extensively studied [12], the association of stress due to inflation and mental health, specifically anxiety and depression, has remained unexplored. There has been some evidence of the association of stress due to inflation with poor mental health in blogs, newspaper articles, and other reports [13,14,15,16].

However, to our knowledge, only one scientific study [7] has considered the relationship between inflation and mental health with a focus on how hardships due to inflation, in other words coping behaviors, such as purchasing less food or delaying medical treatment, are associated with psychological distress. This paper extends this research by considering how self-reported stress due to inflation, irrespective of whether any hardships are experienced, correlates with anxiety and depression.

It is important to examine the association of stress due to inflation with mental health, specifically anxiety, and depression, among working-age adults (18–64 years), because inflationary pressures may disproportionately affect this group due to multiple competing financial obligations, such as those for education, child-care, career investments, home purchases, or stagnant earnings [5]. In addition, working-age adults are probably witnessing the highest inflation of their lifetimes [17] and may not cope well with inflationary pressures.

Inflation is a macro-economic issue that affects the economic security of households. We frame the relationship between inflation and mental health through the biopsychosocial model of health [18]. We situate inflation as one of the social factors that contribute to the development of mental health problems, in addition to, and in interaction with, biological factors (genetics, brain chemistry) and psychological factors (thoughts, emotions, behaviors). Furthermore, we note that stress due to inflation is a potential mechanism whereby inflation may contribute to depression and anxiety. Using the framework by Baum et al (1999) [19], linking the experience of stress and health outcomes, the contribution of stress due to inflation to depression and anxiety is indirect and occurs through one or more of the following channels: (i) biological changes in endocrine and/or immune systems, (ii) behaviors that impact health (e.g. drinking, exercising), and (iii) changes in illness-related behavior (e.g. prevention-related behavior). Socioeconomic status (SES) factors can make the contribution of stress due to inflation to negative changes in behaviors and biological changes worse, for example among persons living in more deprived environments (e.g., communities with limited support structures), with more challenging social conditions (e.g., subject to discrimination), and with other SES factors that affect health, such as limited access to health care or quality nutrition (Baum et al 1999). We, therefore, hypothesize that stress due to inflation, as well as mental distress, will be more prevalent among individuals who are disadvantaged in terms of SES, such as persons who are poor, food insecure, have recently lost employment income, or do not have health insurance.

**Figure 1:**
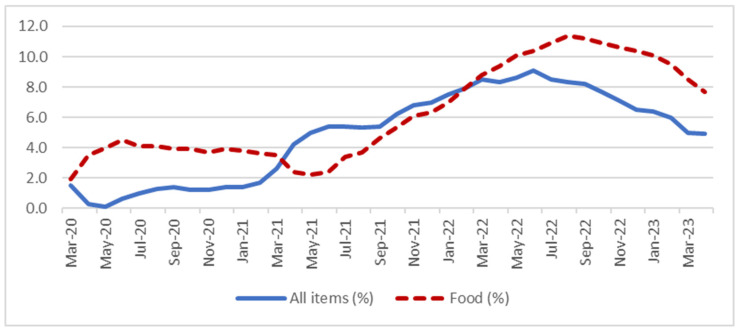
The 12-month percentage change in the Consumer Price Index, all items and food. Source: US Bureau of Labor, https://www.bls.gov/charts/consumer-price-index/consumer-price-index-by-category-line-chart.htm (accessed on 2 May 2023).

We analyzed the association of stress due to inflation with anxiety and depression using cross-sectional data from two time periods (October 2022 and May 2023) of the US Census Bureau HPS. We selected a repeated cross-sectional analysis approach, as there has been a decline in general inflation in recent months, and this may have affected stress levels and their relationships with anxiety and depression. Although one can expect reports about a decline in the general inflation rate in the US to lead to lower rates of stress, food and housing prices have not declined at the same rate as general inflation (Figure 1). The persistent higher food and housing prices compared to pre-COVID-19 negatively affects households and families’ everyday lives. Such ongoing economic hardship without corresponding wage increases can lead to a continued, or even elevated, prevalence of stress over time. With the recent surge and subsequent decline in the inflation rate in the US, there could be significant changes in the prevalence of inflation-related stress and its association with mental health. Therefore, the primary objective of this study is to investigate the prevalence of stress due to inflation during the declining inflation period, and the varying associations of stress due to inflation with anxiety and depression over time among working-age adults in the US.

## 2. Materials and Methods

### 2.1. Study Design

We conducted a pooled cross-sectional analysis of data from the online Household Pulse Survey (HPS), collected during two time periods: 5–17 October 2022 (week 50), and 26 April–8 May 2023 (Week 57). Samples are drawn from the US Census Bureau’s Master Address File in combination with its Contact Frame and contacted via email and text.

### 2.2. Data Source

The HPS is a nationally representative survey conducted by the US Census Bureau in partnership with 16 other federal agencies to collect timely data on the social and economic impacts of the COVID-19 pandemic. It is designed to produce national, state, and metro-level estimates of household experiences [20]. The survey collects information on a range of topics, including socio-demographic characteristics, employment status, food security, healthcare accessibility, physical and mental health, and COVID-19-related information, including vaccines, testing, and symptoms [20]. In October 2022 (Week 50), the HPS included information on how stressful the increase in prices in the last 2 months in areas where the respondents live or shop, which were categorized as very/moderately/a little/not at all stressful.

### 2.3. Study Sample

The study sample consisted of working-age adults (18–64 years of age) who responded that prices had increased in the areas they live in the past two months. Respondents with missing data on depression, anxiety, or stress due to inflation were excluded. The study included 24,150 and 33,262 working-age adults in week 50 and week 57, respectively, representing 75,166,217 and 73,280,979 working-age adults in the US.

### 2.4. Dependent Variables

The study examined two distinct dependent variables: anxiety and depression. The level of anxiety was assessed using the Generalized Anxiety Disorder (GAD-2) scale, with a threshold set at a score of three or higher to indicate the presence of anxiety within the individual’s experience [21]. Additionally, the HPS collected data on the Patient Health Questionnaire (PHQ-2) from the participants. In order to identify instances of depression, the PHQ-2 scores were categorized using a cutoff point of three, designating scores at or above this threshold as indicative of the presence of depression [22].

### 2.5. Key Explanatory Variables (Stress Due to Inflation and Time)

The key explanatory variable is stress due to inflation, which was measured using the following question: How stressful, if at all, has the increase in prices in the last 2 months been for you? Stress due to inflation (or “stress” thereafter) was dichotomized into (1) Very stressful’ and ‘Moderately stressful’ and (2) ‘A little stressful’ and ‘Not at all stressful’. For this paper, “stress due to inflation” will be referred as “stress”.

As we are interested in potential changes over time in the association between stress due to inflation and mental health outcomes, two other key explanatory indicators are the binary indicators for the time periods: 5–17 October 2022 (week 50) versus 26 April–8 May 2023 (Week 57). We combined the binary time indicator and stress due to inflation indicator to group the participants into four categories: (1) ‘Week 50 + Stress’; (2) ‘Week 57 + Stress’; (3) ‘Week 57 + No Stress’; and (4) ‘Week 50 + No Stress’. In statistical analyses, ‘Week 50 + No Stress’ was used as the reference group.

### 2.6. Other Explanatory Variables

We incorporated demographic variables (age and gender), race/ethnicity, social determinants of health (education, employment, lost income (a loss of employment income by individual or family member in the last 4 weeks), author(s) calculated poverty status based on the Federal Poverty Line (FPL), difficulty in meeting household expenses, food insecurity, marital status, and region). To calculate poverty status, income categories were converted to continuous values using a uniform distribution. The FPL was obtained from the ASPE official source: https://aspe.hhs.gov/poverty/figures-fed-reg-htm. Household incomes were then expressed as a percentage of the FPL using the uniform method, leading to the following categorization: Poor (<100% FPL), Near Poor (100–<200% FPL), Middle Income (200–400% FPL), and High Income (>400% FPL). Difficulty in meeting household expenses (no difficulty/little/moderate/very difficult) in the last 7 days was also included, as it is related to mental health problems [23]. We also adjusted for the long COVID and COVID-19 vaccinations, as research demonstrated their effects on mental health [24,25]. Random imputation was used to impute missing data on gender.

### 2.7. Statistical Analysis

The association of time periods with stress due to inflation, and the associations of stress with anxiety and depression were tested using Rao–Scott chi-square tests. Unadjusted logistic regressions were used to identify the association of time period with stress due to inflation for each characteristic of the subgroups. For example, for each gender (males, females, transgender) the association of time with stress was tested. Multivariable logistic regressions were used to analyze the adjusted associations of time, stress due to inflation, and the interaction of stress and time with anxiety and depression. In these regressions, we controlled for age, gender, race and ethnicity, poverty status, food insecurity, education, employment, lost income, difficulty meeting expenses, long COVID, COVID-19 vaccination status, marital status, and region. For explanatory variables with missing data, the missing indicators were used in the logistic regressions. We also used time-stratified multivariable logistic regressions to analyze the association of stress due to inflation with anxiety and depression in October 2022 and May 2023. In the tables, we report the unadjusted odds ratios (UOR), adjusted odds ratios (AOR) and 95% confidence intervals (CI). All analyses were conducted using the SAS survey procedure [26], which incorporated the replicate survey weights provided by the HPS. We also employed a jackknife approximation to adjust for variability in the estimation of these weights [27].

## 3. Results

Overall, 47% of working-age adults were females, and 2.8% reported being transgender. Working-age adults were 19% Latino and 10.6% Black/African American. Over half (54.4%) were currently married; 33.4% had college degrees; and 23.4% were 55–64 years of age (data not presented in tabular form).

The distribution of characteristics did not differ across Weeks 50 and 57 in age group, gender, race and ethnicity, marital status, food insecurity, health insurance coverage, and region (Table S1). However, the representation of Latinos (18.2% in week 50 vs. 19.7% in week 57) and Asians (5.6% vs. 6.1%) increased over time. The proportion of individuals never diagnosed with COVID-19 decreased from 47.7% in Week 50 to 40.5% in Week 57 (Table S1).

### 3.1. Stress Due to Inflation 

78.7% of working-age adults reported experiencing stress due to inflation in May 2023, compared to 77.7% in October 2022 (UOR= 1.06 95% CI = 1.00, 1.13; *p* = 0.033 (Table 1). Among individuals with food insecurity, the prevalence of stress due to inflation was very high and remained at 98% (Table 1) in both time periods. Similarly, the prevalence of stress among working-age adults with incomes below 100% FPL (89.6% vs. 88.7%) and those who reported lost income from employment in their household during the past four weeks remained very high (92%).

The prevalence of stress due to inflation significantly increased over time for specific subgroups (Table 1). For example, those finding it very difficult to meet expenses (97.9% vs. 99.1%) experienced a significant increase in stress due to inflation (Table 1). Non-Hispanic White (74.7% vs 76.2% (UOR, 95% CI = 1.09 [1.02, 1.16]), employed (76.1% vs. 77.6% (UOR [95% CI] = 1.09 [1.02,1.16]), middle income (UOR [95% CI] = 1.28 [1.14, 1.43]), and food secure individuals (UOR [95% CI] = 1.07 [1.01, 1.14]) were also more likely to report stress in May 2023 in comparison to October 2022 (Table 1).

### 3.2. Anxiety and Depression

Among all working-age adults, the prevalence of anxiety decreased by three percentage points from October 2022 to May 2023—35.4% vs. 32.1% (Table 2). In the fully adjusted logistic regression, working-age adults were less likely to report anxiety in May 2023 compared to October 2022 (AOR 95% CI = 0.86 [0.80, 0.94] (Table 3). Similarly, the prevalence of depression decreased by three percentage points from October 2022 to May 2023—25.5% vs. 22.6% (Table 2). In the fully adjusted logistic regression, working-age adults were less likely to report depression in May 2023 compared to October 2022 (AOR =0.85; 95% CI = 0.78, 0.93 (Table 3).

As we are interested in the relationship of stress with anxiety and depression, we also compared the prevalence of anxiety and depression by week among the stress groups. There was a significant decline in the prevalence of anxiety in working-age adults with (41.8% vs. 37.6%) and without stress (12.9% vs. 11.9%) (Table 2). A fully adjusted logistic regression with interaction of stress with time period confirmed these findings. For example, those with stress in May 2023 were less likely to report anxiety compared to those with stress in October 2022 (AOR = 0.86 95% CI = 0.79, 0.93—results not reported in tabular form). Similarly, the prevalence of depression among individuals with stress due to inflation also declined from 34.1% in October 2022 to 29.8% in May 2023 (Table 2), and stressed individuals were less likely to report depression in May 2023 in comparison to October 2022, with AOR = 0.83 and 95% CI = 0.76, 0.91.

### 3.3. Pooled Cross-Sections-Multivariable Logistic Regression

In multivariable logistic regressions with pooled cross-sections, the odds of having anxiety or depression were more than twice as high among working-age adults with stress due to inflation compared to adults without stress (Table 3). More precisely, working-age adults with stress due to inflation were more likely to have anxiety (AOR = 2.50, 95% CI = 2.18, 2.86) and to have depression (AOR = 2.22, 95% CI = 1.92, 2.57) compared to those without stress (Table 3).

When the stress variable was interacted with time, we found that individuals with stress were more likely to have anxiety in week 50 (AOR = 2.69, 95% CI = 2.22, 3.26), as well as week 57 (AOR = 2.29, 95% CI = 1.88, 2.77), in reference to individuals with no stress due to inflation in week 50 (Table 3). A similar pattern was observed for depression (AOR = 2.48, 95% CI = 2.03, 3.03 for individuals with stress in week 50; AOR = 2.05, 95% CI = 1.68, 2.50 for individuals with stress in week 57 in reference to individuals with no stress in week 50) (Table 3). However, for both anxiety and depression, the confidence intervals of the interaction terms for stress and week overlap for week 50 and week 57. The descriptive result from Table 2 that anxiety and depression may be less prevalent in week 57 than in week 50 is, therefore, not confirmed by the fully adjusted regressions with interaction terms.

### 3.4. Stratified Multivariable Logistic Regression

We analyzed the associations of stress with anxiety and depression over time with time-stratified multivariable logistic regressions. Stress was positively associated with anxiety and depression in both time periods (Table 3). For anxiety, the AOR was 2.64 (95% CI = 2.15, 3.23) in October 2022, and 2.26 (95% CI = 1.93, 2.66) in May 2023. A similar pattern was observed for depression, with AOR = 2.53, 95% CI = [2.04, 3.14] in October 2022, and AOR = 1.90, 95% CI = [1.57, 2.31] in May 2023.

## 4. Discussion

The present study examined the prevalence of stress due to inflation and its association with anxiety and depression between October 2022 and May 2023 among working-age adults in the US using Census HPS data. Stress due to inflation is common, affecting more than three quarters of working-age Americans. We observed that, despite declining inflation rates, the prevalence of stress due to inflation among working-age adults increased by one percentage point over time, and the increase was statistically significant, thus affecting two million additional working-age adults.

The prevalence of stress was higher among individuals in disadvantaged SES situations in both time periods, such as individuals who reported difficulty in paying expenses or poor and low-income individuals. Some subgroups, however, showed some changes over time. For example, the middle-income group experienced an increase in stress due to inflation level over time. This persistent and increased stress over time among working-age adults grappling with economic hardship can be attributed, in part, to the unprecedented inflationary conditions at the time, possibly the highest inflation of their lifetimes [17]. It is important to note that, while the overall inflation rate may have diminished, price levels remained high, and the reduction in inflation concerning essential commodities, particularly food, has been significantly slower (Figure 1). This sluggish decline in food-related inflation has resulted in persistently high food prices, further exacerbating the economic strain faced by working-age individuals, including professional advancement and family responsibilities [5,28].

Stress due to inflation was associated with depression and anxiety in both time points. Previous research has consistently demonstrated the detrimental impact of macroeconomic factors, such as recessions, on mental health, with increased rates of anxiety, depression, and even suicidal thoughts [29,30,31]. Moreover, at an individual level, a comprehensive review revealed a strong association between financial hardship and depression [11]. These findings emphasize the significant influence of economic conditions on mental well-being, and underscore the need for targeted interventions and support during times of financial strain.

Overall, although stress due to inflation persisted over time, the prevalence of anxiety and depression was lower by 3 percentage points in May 2023 compared to October 2022, and the decline was statistically significant (*p* = 0.033). This decline could be partially explained by the differences in some characteristics between October 2022 and May 2023. For example, there was an increase in rates of employment and a decrease in the poverty rate in May 2023 compared to October 2022. Furthermore, the proportion of individuals expressing difficulty in paying expenses has declined (Table S1). Given that these factors have demonstrated significant associations with anxiety and depression [32], this may suggest that individuals are coping and/or adjusting to their stressful situations [33], or that individuals had more access to food banks or the Supplemental Nutritional Assistance Program (SNAP), with an increased use of food stamps over the past several years [34]. Our descriptive results suggest that, when examined by stress levels, the prevalence of anxiety and depression might have weakened over time for stressed individuals. With increased experience and exposure to handle such stress, individuals may become more adept at navigating challenges and maintaining emotional well-being [35]. To investigate the challenges posed by rising inflation, the HPS included several questions on coping strategies during both Week 50 and Week 57. Individuals experiencing stress employed a diverse array of coping strategies (ranging from 82.0% to 96.6% in October 2022, and 83.3% to 97.4% in May 2023) when confronting inflationary pressures, as opposed to those not experiencing stress (with a range of 3.4% to 18.0% in October 2022, and 2.6% to 16.7% in May 2023). The participants in April–May 2023 drove less or changed their mode of transportation as compared with the participants in October 2022; they were also more likely to shop at stores offering lower prices, look for sales, and use coupons. There was an increase in the proportion of respondents who canceled or reduced their magazine/cable subscriptions and worked additional jobs in April–May 2023 as compared with October 2022. These strategies indicated that efforts were being made to adjust to the inflationary environment. Apart from the coping strategies, there were more participants in April–May 2023 than in October 2020 who ate out less frequently (Table S2). These findings underscore the relationship of stress due to inflation with individuals’ financial and mental well-being.

The strength of this study lies in its comprehensive examination of the association of stress due to inflation with anxiety and depression among working-age adults. By comparing data from the HPS for two periods, the study provides valuable insights into the persistence of stress due to inflation and of its association with anxiety and depression during a time of post-pandemic recovery with declining inflation. Additionally, the study incorporates various demographic and socioeconomic factors, such as age, gender, income, and employment status, allowing for a more nuanced understanding of the complex relationship between stress due to inflation and mental health outcomes. The large sample size and statistical analysis further enhance the study’s validity and the generalizability of the findings to the broader population. While an earlier study [7] had shown an association between inflation hardships (e.g., purchasing less food, working additional jobs) and mental health problems, this study shows that stress due to inflation is associated with anxiety and depression, and may be a mechanism whereby inflation impacts health, whether or not individuals experience hardships.

Despite its strengths, this study has certain limitations that should be considered. Firstly, the data used in the analysis relies on self-reported responses from survey participants, which may be subject to recall bias or social desirability bias. Additionally, the study compared the most recent HPS survey (week 57) at the time the analysis was conducted with October 2022 (Week 50), when inflation was approximately 7%, not to the peak in July 2022, when inflation was 9%. HPS may not capture the long-term effects of stress due to inflation on mental health. The cross-sectional design of the study limits the ability to establish causal relationships between stress due to inflation and anxiety/depression. We use cross-sectional data with no information on the onset of anxiety and depression and whether it may have predated stress due to inflation. Further research on stress due to inflation and mental health would benefit from the use of longitudinal data. While stress due to inflation may adversely impact mental health, anxiety and depression may make individuals more likely to perceive and report stress due to inflation. Finally, there may be confounding factors that may affect both self-perceived stress and mental health, such as the presence or absence of social support networks, which may influence the observed association. Finally, the study primarily focuses on working-age adults and does not capture the experiences and mental health outcomes of older adults.

The study’s findings suggest the need for interventions to promote financial resilience to help individuals manage their finances during economic fluctuations and reduce stress due to inflation. In addition, greater access to affordable mental health services may help address stress due to inflation. Policies are needed to expand such support with a focus on underserved communities with culturally relevant care. Addressing systemic disparities, such as income inequality and access to mental health care, is crucial. Long-term monitoring and research on stress due to inflation are needed to understand its effects and develop interventions to reduce it. Collaboration among policymakers, healthcare providers, and community organizations is essential for implementing these recommendations and supporting affected individuals and communities.

## 5. Conclusions

In conclusion, stress due to inflation affects more than three-quarters of working-age adults in the US. Despite declining inflation, stress due to inflation persists, and continues to be associated with anxiety and depression. Stress due to inflation is a significant and persistent public health issue.

## Figures and Tables

**Table 1:** Prevalence of stress due to inflation among working-age adults over time, according to the Household Pulse Survey (–14 October 22022 and 26 April–8 May 2023).

Variable	Stress Due to Inflation (Week 50)		Stress Due to Inflation (Week 57)		UOR	95% CI	*p*-Value
	Number	Row Wt%	Number	Row Wt%			
All	17,365	77.7	24,314	78.7	1.06	[1.00, 1.13]	0.033
Difficulty in Paying Expenses							
Not Difficult	3174	41.0	4405	43.0	1.09	[0.99, 1.20]	0.088
Little Difficult	5899	84.0	8403	84.5	1.04	[0.89, 1.21]	0.606
Somewhat Difficult	4504	92.8	6265	94.4	1.31	[1.02, 1.69]	0.034
Very Difficult	3782	97.9	5221	99.1	2.24	[1.05, 4.80]	0.036
Gender							
Female	9866	81.0	14,736	81.4	1.03	[0.95, 1.12]	0.441
Male	7089	74.0	9041	75.4	1.08	[0.98, 1.19]	0.139
Transgender	410	79.3	537	86.4	1.65	[0.81, 3.36]	0.166
Age							
18–34 years	4251	78.4	5508	80.3	1.12	[1.00, 1.27]	0.054
35–44 years	4210	77.0	6470	78.5	1.09	[0.95, 1.25]	0.234
45–54 years	4185	79.9	6021	79.9	1.00	[0.87, 1.14]	0.969
55–64 years	4719	75.3	6315	75.6	1.02	[0.90, 1.15]	0.741
Race and Ethnicity							
NHW	12,721	74.7	17,111	76.2	1.09	[1.02, 1.16]	0.011
NHB	1226	80.9	1951	80.2	0.96	[0.76, 1.21]	0.724
Hispanic/Latino	1839	85.4	2867	86.2	1.07	[0.81, 1.41]	0.630
Asian	726	75.5	1161	71.6	0.82	[0.62, 1.09]	0.161
Other Race	853	81.2	1224	83.1	1.14	[0.82, 1.59]	0.438
Marital Status							
Married	9324	75.7	13,136	75.8	1.00	[0.93, 1.09]	0.914
Widowed	438	87.6	579	88.0	1.04	[0.63, 1.70]	0.883
Separated	2675	85.6	3825	84.7	0.93	[0.73, 1.18]	0.542
Divorced	403	90.9	549	92.2	1.17	[0.60, 2.28]	0.636
Never Married	4480	76.5	6153	80.2	1.24	[1.07, 1.44]	0.004
Education							
Less than High School	417	88.8	611	88.3	0.95	[0.54, 1.66]	0.849
High School	2358	83.2	3498	85.9	1.23	[1.00, 1.50]	0.043
Some College	4298	82.2	5807	84.5	1.18	[1.01, 1.38]	0.042
Associate Degree	2137	83.9	2881	80.5	0.79	[0.63, 0.98]	0.033
College	8155	65.8	11,517	66.4	1.03	[0.96, 1.11]	0.436
Employment							
Employed	12,865	76.1	18,561	77.6	1.09	[1.02, 1.16]	0.007
Not Employed	4449	81.6	5666	82.0	1.03	[0.88, 1.20]	0.712
Lost Income							
Yes	2165	92.2	2940	92.1	0.99	[0.73, 1.33]	0.926
No	15,170	75.3	21,331	76.7	1.08	[1.02, 1.15]	0.012
Poverty Status (based on FPL)							
Poor	2126	89.6	2629	88.7	1.12	[0.80, 1.56]	0.498
Low Income	3086	86.9	4156	88.6	0.97	[0.74, 1.28]	0.848
Middle Income	5122	81.1	7081	84.1	1.28	[1.14, 1.43]	<0.001
High Income	5728	61.3	8655	62.5	1.05	[0.95, 1.15]	0.342
Food Insecurity							
Food Insecure	2179	98.0	3071	98.3	1.18	[0.65, 2.12]	0.585
Food Secure	15,137	74.4	21,164	75.7	1.07	[1.01, 1.14]	0.031
Health Insurance							
Yes	15,904	76.6	22,343	77.5	1.05	[0.99, 1.12]	0.084
No	1187	87.3	1553	88.1	1.08	[0.73, 1.59]	0.704
Region							
Northeast	2307	76.9	3384	76.0	0.95	[0.81, 1.12]	0.528
South	5675	79.2	8010	81.4	1.15	[1.03, 1.29]	0.012
Midwest	3837	74.4	5218	76.8	1.14	[1.03, 1.27]	0.012
West	5546	78.6	7702	77.7	0.95	[0.83, 1.08]	0.413
COVID-19 Vaccine							
Yes	14,077	75.0	19,820	76.9	1.11	[1.04, 1.18]	0.001
No	3218	88.0	4345	86.2	0.85	[0.65, 1.11]	0.227
Long COVID							
Long COVID	3058	86.9	4812	86.0	0.93	[0.74, 1.17]	0.544
Short COVID	5945	73.6	9710	74.2	1.04	[0.94, 1.14]	0.475
No COVID	8276	77.3	9640	80.0	1.17	[1.05, 1.31]	0.004

Based on 57,412 working-age adults (age 18–64 years) and those who said prices have increased, the table presents the prevalence of stress due to inflation for various characteristics. Row percentages of stress due to inflation were calculated for week 50 and week 57, separately. There is no missing data on stress due to inflation, anxiety, and depression variables. Missing data in difficulty in paying for household expenses, marital status, employment, lost income from employment, poverty, food insecurity, private health insurance, COVID-19 vaccine, long COVID are not included in the table. Table 1 includes the unadjusted odds ratio and a 95% confidence interval from a logistic regression of week number (week 57 vs week 50 (reference)) on stress due to inflation for each subgroup. UOR: unadjusted odds ratio; CI: confidence interval COVID: coronavirus disease; NHB: non-Hispanic Black; NHW: non-Hispanic White; Wt: weighted; FPL: federal poverty line.

**Table 2:** Description of working-age adults (age 18–64 years) with anxiety and depression (row percentages) Census Household Pulse Survey, 2–14 October 2022 (Week 50) and 26 April–8 May 2023 (Week 57).

Variable	With Anxiety			With Depression		
	N	wt%	*p*-Value	N	wt%	*p*-Value
ALL	17,891	33.8		12,995	27.0	
Week Number			<0.001			<0.001
Week 50	7884	35.4		5796	28.7	
Week 57	10,007	32.1		7199	25.4	
Stress of Price Increase			<0.001			<0.001
Stressed	15,988	39.7		11,751	31.9	
No Stress	1903	12.4		1244	9.5	
Week and Stress due to Inflation			<0.001			<0.001
Week 50 + Stress	7023	41.8		5211	34.1	
Week 50 + No Stress	861	12.9		585	9.9	
Week 57 + Stress	8965	37.6		6540	29.8	
Week 57 + No Stress	1042	11.9		659	9.1	
Difficulty in Paying Expenses			<0.001			<0.001
Very Difficult	5891	62.3		4741	53.6	
Somewhat Difficult	4624	39.4		3320	31.8	
Little Difficult	4549	26.2		3142	19.6	
Not Difficult	2817	15.4		1784	11.0	
Gender			<0.001			<0.001
Female	10,962	36.1		7413	26.7	
Male	6269	29.6		5051	25.5	
Transgender	660	61.5		531	57.3	
Age			<0.001			<0.001
18–34 years	5131	41.5		3823	34.5	
35–44 years	4668	33.1		3193	24.7	
45–54 years	4167	31.3		2991	24.4	
55–64 years	3925	26.2		2988	21.7	
Race and Ethnicity			<0.001			<0.001
NHW	13,186	34.8		9316	26.8	
NHB	1213	32.3		991	27.6	
Hispanic/Latino	1919	33.4		1452	28.3	
Asian	560	21.9		469	19.2	
Other Race	1013	39.8		767	32.4	
Marital Status			<0.001			<0.001
Married	8362	28.2		5491	20.4	
Widowed	434	40.9		365	38.1	
Separated	2950	38.4		2257	32.1	
Divorced	488	41.4		399	35.2	
Never Married	5616	41.1		4451	35.9	
Education			<0.001			<0.001
Less than High School	476	37.7		418	34.1	
High School	2508	35.4		2129	31.4	
Some College	4694	39.8		3652	32.9	
Associate Degree	2178	37.1		1645	29.2	
College	8035	26.7		5151	17.5	
Employment			<0.001			<0.001
Employed	12,949	31.6		9044	24.6	
Not Employed	4900	39.8		3918	33.8	
Lost income			<0.001			<0.001
Yes	2935	51.6		2318	42.5	
No	14,931	31.1		10,658	24.7	
Poverty			<0.001			<0.001
Poor	2585	45.5		2173	41.5	
Low Income	3521	40.7		2748	34.1	
Middle Income	5117	33.9		3684	26.2	
High Income	5449	23.7		3525	16.5	
Food Insecurity			<0.001			<0.001
Low/Very Low	3524	62.5		3042	56.4	
Food Secure	14,325	29.3		9918	22.5	
Health Insurance			<0.001			<0.001
Yes	16,156	32.8		11,558	25.8	
No	1440	44.5		1221	40.5	
Region			0.475			0.036
Northeast	2425	33.0		1679	25.4	
South	5870	34.3		4310	28.2	
Midwest	3843	33.1		2821	26.4	
West	5753	34.1		4185	26.9	
COVID-19 Vaccine			<0.001			<0.001
Yes	14,763	32.7		10,558	25.8	
No	3063	38.4		2388	32.4	
Long COVID			<0.001			<0.001
Long COVID	4282	47.4		3257	37.3	
Short COVID	5803	27.2		3879	20.6	
No COVID	7731	34.3		5805	28.9	
Comorbid Depression			<0.001			
Yes	10,712	82.0				
No	7179	15.9				
Comorbid Anxiety						<0.001
Yes				10,712	65.7	
No				2283	7.4	

Notes: Based on 57,412 working-age adults (age 18–64 years) and those who said prices have increased. No missing data on stress due to inflation, anxiety, and depression variables. Missing data in difficulty in paying for household expenses, marital status, employment, lost income from employment, income, food insecurity, private health insurance, COVID-19 vaccine, long COVID are not included in the table. Rao–Scott chi-squared test was used to determine significant group differences in anxiety and depression. Except for the region, all categories are highly significant. COVID: coronavirus disease; NHB: non-Hispanic Black; NHW: non-Hispanic White; Wt: weighted.

**Table 3:** Unadjusted odds ratios (UOR) and adjusted odds ratios (AOR) and 95% confidence intervals (CI) from separate logistic regressions on anxiety and depression in working-age adults (age 18–64 years) according to data from the Census Household Pulse Survey, 2–14 October 2022, and 26 April–8 May 2023.

Dependent Variable = Anxiety						
Logistic Regressions	Model 1 ^#^			Fully Adjusted Models		
	AOR	95% CI	*p*-Value	AOR	95% CI	*p*-Value
**Week**						
Week 57	0.85	[0.79, 0.91]	<0.001	0.86	[0.80, 0.94]	<0.001
Week 50 (Ref)						
**Stress due to Inflation**						
Stress	4.68	[4.24, 5.17]	<0.001	2.50	[2.18, 2.86]	<0.001
No Stress (Ref)						
**Logistic Regressions with Interaction Terms**	**UOR**	**95% CI**	***p*-value**	**AOR**	**95% CI**	***p*-value**
**Week and Stress Interaction**						
Week 50 + Stress	4.87	[4.21, 5.63]	<0.001	2.69	[2.22, 3.26]	<0.001
Week 57 + Stress	4.08	[3.53, 4.71]	<0.001	2.29	[1.88, 2.77]	<0.001
Week 57 + No Stress	0.91	[0.76, 1.09]	0.324	1.02	[0.83, 1.25]	0.860
Week 50 + No Stress (Ref)						
**Logistic Regressions Stratified by Week Number**	**UOR**	**95% CI**	***p*-value**	**AOR**	**95% CI**	***p*-value**
**Week 50: Stress due to Inflation**						
Stress	4.87	[4.21, 5.63]	<0.001	2.64	[2.15, 3.23]	<0.001
No Stress (Ref)						
**Week 57: Stress due to Inflation**						
Stress	4.47	[3.91, 5.11]	<0.001	2.26	[1.93, 2.66]	<0.001
No Stress (Ref)						
**Dependent Variable = Depression**						
	**Model 1 ^#^**			**Fully Adjusted Models**		
**Logistic Regressions**	**AOR**	**95% CI**	***p*-Value**	**AOR**	**95% CI**	***p*-Value**
**Week**						
Week 57	0.83	[0.76, 0.90]	<0.001	0.85	[0.78, 0.92]	<0.001
Week 50 (Ref)						
**Stress due to Inflation**						
Stress	4.48	[3.86, 5.19]	<0.001	2.22	[1.92, 2.57]	<0.001
No Stress (Ref)						
**Logistic Regressions with Interaction terms**	**UOR**	**95% CI**	***p*-value**	**AOR**	**95% CI**	***p*-value**
**Week and Stress Interaction**						
Week 50 + Stress	4.70	[3.70, 5.96]	<0.001	2.48	[2.03, 3.03]	<0.001
Week 57 + Stress	3.85	[3.04, 4.88]	<0.001	2.05	[1.68, 2.50]	<0.001
Week 57 + No Stress	0.91	[0.69, 1.20]	0.517	1.06	[0.84, 1.33]	0.634
Week 50 + No Stress (Ref)						
**Logistic Regressions Stratified by Week Number**	**UOR**	**95% CI**	***p*-value**	**AOR**	**95% CI**	***p*-value**
**Week 50: Stress due to Inflation**						
Stress	4.70	[3.70, 5.96]	<0.001	2.53	[2.04, 3.14]	<0.001
No Stress (Ref)						
**Week 57: Stress due to Inflation**						
Stress	4.22	[3.57, 4.99]	<0.001	1.90	[1.57, 2.31]	<0.001
No Stress (Ref)						

Notes: Based on 57,412 working-age adults (age 18–64 years) and those who reported prices have increased and had no missing data on stress due to price increase, anxiety, and depression variables. **^#^** Model 1 adjusted for week number and stress due to inflation. Stratified and fully adjusted logistic regressions controlled for the following additional variables: difficulty in paying for household expenses, gender, race and ethnicity, age, education, employment, lost income from employment, poverty based on federal poverty line, food insecurity, private health insurance, marital status, region, COVID-19 vaccine and long COVID. Ref: Reference group.

## Data Availability

No restrictions apply to the availability of these data. Data were obtained from the US Census Bureau, and are available at https://www.census.gov/programs-surveys/household-pulse-survey/datasets.html (accessed on 11 September 2024).

## Supplementary Materials

The following supporting information can be downloaded at: https://www.mdpi.com/article/10.3390/ijerph22010026/s1, Table S1: title; Description of Working-Age Adults (Age 18–64 years) by Week of Participation in the Census Household Pulse Survey, 2–14 October 2022 (Week 50) and April 26–May 8, 2023 (Week 57); Table S2: Title: Coping Mechanisms for Stress due to Inflation by Stress Level in Census Household Pulse Survey Respondents 2–14 October 2022 (Week 50) and 26 April –8 May 2023 (Week 57).
