# Supplementary material for: Stress Due to Inflation and Its Association with Anxiety and Depression Among Working-Age Adults in the United States
Source: Int J Environ Res Public Health. 2024 Dec 29;22(1):26. doi: 10.3390/ijerph22010026 (PMC11764509; doi:10.3390/ijerph22010026)
Supplement: Supplementary file 1 [file ijerph-22-00026-s001.zip › ijerph-3294199 supplementary.pdf]

Table S1. Description of working-age adults (age 18-64 years) by week of participation, according to data from the Census Household Pulse Survey, 2–14 October 2022 (Week 50) and 26 April–8 May 2023 (Week 57).

	**Week 50**		**Week 57**		
	**2–14 October 2022**		**26 April–8 May 2023**		***p*-Value**
	**N**	**Col wt %**	**N**	**Col wt %**	
ALL	24,150	100.0	33,262	100.0	
Stress of Price Increase					0.033
Very/Moderate Stress	17,365	77.7	24,314	78.7	
Little/ No Stress	6785	22.3	8948	21.3	
Gender					0.348
Female	13,186	50.5	19,411	49.9	
Male	10,469	46.5	13,202	47.4	
Transgender	495	3.0	649	2.7	
Age					0.067
18-34 years	5798	31.2	7279	32.2	
35-44 years	5881	22.8	8832	23.2	
45-54 years	5696	22.1	8189	21.7	
55-64 years	6775	23.9	8962	23.0	
Race and Ethnicity					0.003
NHW	18,121	60.8	23,901	58.4	
NHB	1583	10.4	2536	10.8	
Hispanic / Latino	2251	18.2	3505	19.7	
Asian	1089	5.6	1794	6.1	
Other race	1106	5.0	1526	5.0	
Education					0.005
Less than High School	469	6.2	709	7.3	
High School	2819	28.8	4089	28.5	
Some College	5257	22.0	6968	20.9	
Associate Degree	2612	9.9	3543	9.5	
College	12,993	33.1	17,953	33.7	
Employment					<0.001
Employed	18,380	71.3	25,948	74.8	
Not Employed	5706	28.4	7205	24.9	
Poverty					<0.001
Poor	2330	15.6	2950	14.2	
Low Income	3582	19.0	4724	18.6	
Middle Income	6441	25.2	8740	25.9	
High Income	10,100	30.1	14,6260	32.7	
Food Insecurity					0.091
Low/Very Low	2220	13.7	3129	13.3	
Food Secure	21,869	86.1	30,032	86.2	
Health Insurance					0.794
Yes	22,376	89.0	30,966	89.2	
No	1400	8.6	1800	8.3	
Region					0.010
Northeast	3340	16.7	4800	16.7	
South	7692	37.7	10,687	38.7	
Midwest	5420	21.1	7191	20.0	
West	7698	24.6	10,584	24.6	
Difficulty in Paying Expenses					0.005
Not At All	8378	26.3	11,192	26.4	
Little	7108	29.4	10,096	30.6	
Somewhat	4832	23.6	6665	23.1	
Very Difficult	3825	20.7	5282	19.8	

Notes: Based on 57,412 working-age adults (age 18–64 years) and those who said prices have increased. No missing data on stress due to price increase, anxiety, and depression variables. Missing data in difficulty in

paying for household expenses, marital status, employment, lost income from employment, poverty, food insecurity, private health insurance, COVID-19 vaccine, long COVID are not included in the table. Rao–Scott chi-squared test was used to determine significant group differences by week of participation. Employment, income and long COVID are highly significant. COVID: coronavirus disease; NHB: non-Hispanic Black; NHW: non-Hispanic White; Col Wt: column-weighted percentage.

Table S2. Coping mechanisms for stress due to inflation by stress level in Census Household Pulse Survey respondents between 2–14 October 2022 (Week 50) and 26 April–8 May 2023 (Week 57).

Variable	2–14 October 2022				26 April–May 2023			
	Stress		No Stress		Stress		No Stress	
	Number	wt %	Number	wt %	Number	wt %	Number	wt %
**All**	17,365	77.7	6785	22.3	24,314	78.7	8948	21.3
Shop at stores that offer lower prices, look for sales, and/or use coupons								
Yes	12,306	85.7	2741	14.3	17,742	84.1	4346	15.9
No	5059	62.6	4044	37.4	6572	67.6	4602	32.4
Switch to generic product								
Yes	9730	88.4	1669	11.6	13,615	86.9	2671	13.1
No	7635	67.4	5116	32.6	10,699	70.7	6277	29.3
Purchase less fresh produce and/or meat								
Yes	8063	93.6	791	6.4	9108	93.3	772	6.7
No	9302	67.7	5994	32.3	15,206	72.1	8176	27.9
Less often eating out or order food								
Yes	12,160	83.6	3016	16.4	16,355	83.3	4342	16.7
No	5205	68.7	3769	31.3	7959	72.4	4606	27.6
Cancel/reduce subscriptions								
Yes	8663	88.7	1427	11.3	12,852	88.1	2465	11.9
No	8702	69.8	5358	30.2	11,462	71.1	6483	28.9
Cancel/decrease plans to attend events								
Yes	8856	91.2	1076	8.8	11,739	91.3	1485	8.7
No	8509	68.2	5709	31.8	12,575	70.5	7463	29.5
Drive less/change mode of transportation								
Yes	7209	88.5	1206	11.5	7053	91.1	922	8.9
No	10,156	71.8	5579	28.2	17,261	74.8	8026	25.2
Delay major purchase								
Yes	11,331	88.0	2014	12.0	15,675	87.5	2954	12.5
No	6034	65.3	4771	34.7	8639	68.3	5994	31.7
Delay medical treatment								
Yes	5398	95.1	336	4.9	7762	95.2	466	4.8
No	11,967	71.9	6449	28.1	16,552	72.8	8482	27.2
Work additional jobs								
Yes	4234	91.1	484	8.9	6794	90.8	917	9.2
No	13,131	74.0	6301	26.0	17,520	74.7	8031	25.3
Less savings								
Yes	8414	90.0	1254	10.0	10,839	90.4	1540	9.6
No	8951	70.0	5531	30.0	13,475	72.3	7408	27.7
Increase use of credit card								
Yes	6041	92.7	641	7.3	7186	93.9	591	6.1
No	11,324	71.7	6144	28.3	17,128	73.9	8357	26.1
Decrease use utilities								
Yes	5852	90.3	743	9.7	7689	91.3	974	8.7
No	11,513	72.8	6042	27.2	16,625	74.3	7974	25.7
Move less expensive housing								
Yes	749	93.5	55	6.5	1371	93.2	135	6.8
No	16,616	77.0	6730	23.0	22,943	77.8	8813	22.2
Ask friends/family for help								
Yes	3012	96.6	132	3.4	3468	96.2	175	3.8

No	14,353	74.0	6653	26.0	20,846	76.1	8773	23.9
Change/reduce childcare								
Yes	790	96.5	49	3.5	1134	94.6	94	5.4
No	16,575	76.9	6736	23.1	23,180	78.0	8854	22.0
Utilize benefits from charities								
Yes	1130	95.5	44	4.5	1548	97.4	53	2.6
No	16,235	76.6	6741	23.4	22,766	77.7	8895	22.3
Used other coping mechanism								
Yes	913	82.0	151	18.0	1272	88.6	189	11.4
no	16,452	77.4	6634	22.6	23,042	78.1	8759	21.9

Notes: Based on 57,412 working-age adults (age 18–64 years) and those who said prices have increased. The table presents the prevalence of various coping mechanisms to deal with inflation. Row percentages presented in table of various coping mechanisms were calculated for week 50 and week 57 separately. Wt: Weighted.
